# Digitisation of the Natural History Museum’s collection of *Dalbergia*, *Pterocarpus* and the subtribe Phaseolinae (Fabaceae, Faboideae)

**DOI:** 10.3897/BDJ.10.e94939

**Published:** 2022-11-14

**Authors:** Krisztina Lohonya, Laurence Livermore, Jacek Wajer, Robyn Crowther, Elizabeth Devenish

**Affiliations:** 1 The Natural History Museum, London, United Kingdom The Natural History Museum London United Kingdom

**Keywords:** digitisation, Phaseolinae, *Dalbergia*, *Pterocarpus*, legumes, georeferencing, transcription, crop wild relatives, specimens, rosewoods, cultivated beans, sustainable timber, herbarium, herbaria, natural history collections

## Abstract

**Background:**

In 2018, the Natural History Museum (NHMUK, herbarium code: BM) undertook a pilot digitisation project together with the Royal Botanic Gardens Kew (project Lead) and the Royal Botanic Garden Edinburgh to collectively digitise non-type herbarium material of the subtribe Phaseolinae and the genera *Dalbergia* L.f. and *Pterocarpus* Jacq. (rosewoods and padauk), all from the economically important family of legumes (Leguminosae or Fabaceae).

These taxonomic groups were chosen to provide specimen data for two potential use cases: 1) to support the development of dry beans as a sustainable and resilient crop; 2) to aid conservation and sustainable use of rosewoods and padauk. Collectively, these use case studies support the aims of the UK’s Department for Environment Food & Rural Affairs (DEFRA)-allocated, Official Development Assistance (ODA) funding.

**New information:**

We present the images and metadata for 11,222 NHMUK specimens. The metadata includes label transcription and georeferencing, along with summary data on geographic, taxonomic, collector and temporal coverage. We also provide timings and the methodology for our transcription and georeferencing protocols. Approximately 35% of specimens digitised were collected in ODA-listed countries, in tropical Africa, but also in South East Asia and South America.

## Introduction

The role of natural history collections in conservation science research continues to grow. When correctly identified and interpreted, digitised herbarium specimens can provide important information about the distribution of the individual species and also highlight which species occur naturally together (Canteiro et al. 2019). This information can greatly help with the species conservation assessments, especially for large and diverse genera (Nic Lughadha et al. 2019) or crop wild relatives (Castañeda-Álvarez et al. 2016).

While there have been collaborative efforts between herbaria in the past, these have tended to prioritise digitisation of type specimens (Haston et al. 2012, Borsch et al. 2020). In this project, we piloted collaborative digitisation of ca. 37,000 non-type specimens of the subtribe Phaseolinae and the genera *Dalbergia* L.f. and *Pterocarpus* Jacq. (rosewoods and padauk) from the Royal Botanic Gardens Kew (K), Natural History Museum, London (BM), and the Royal Botanic Garden Edinburgh (E) (Drinkwater 2019, Hirschler 2019).

These taxonomic groups were chosen for two case studies using herbarium collections to support the aims of the UK’s Department for Environment Food & Rural Affairs (DEFRA)-allocated, Official Development Assistance (ODA) funding.

**Figure 1.**
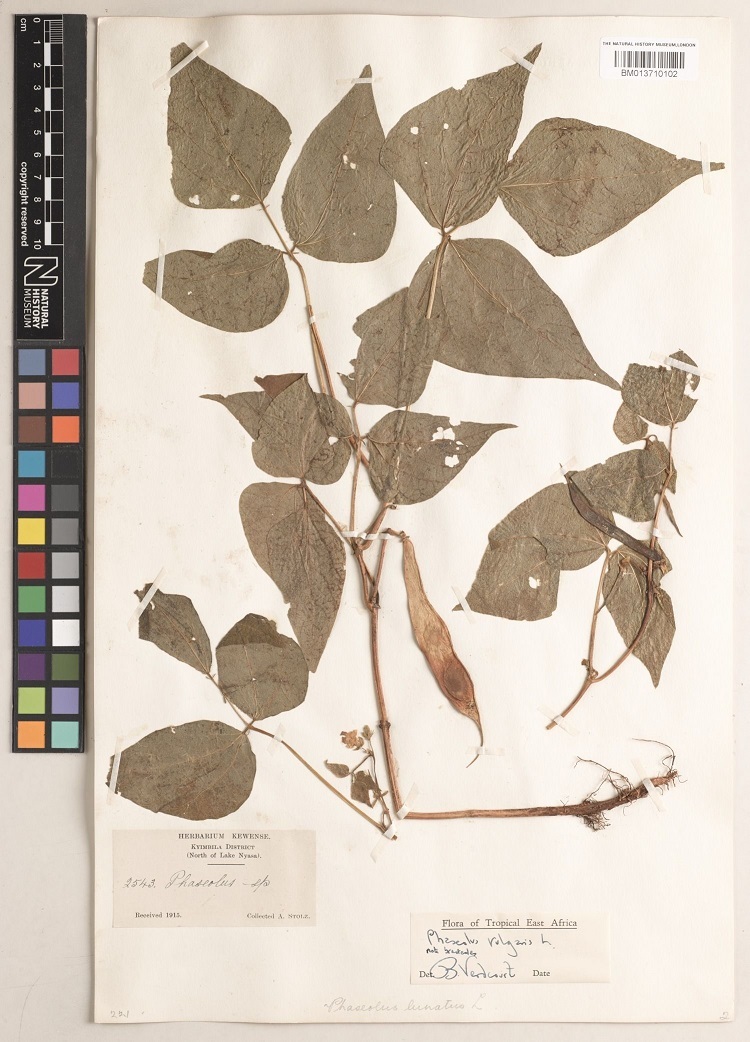
*Phaseolusvulgaris* L. (BM013710102).

The first case study focused on the subtribe Phaseolinae, which includes important food crop plants (Chibarabada et al. 2017). For example, the subtribe has many cultivated beans for humans and livestock, such as: dry beans, (*Phaseolus* spp.) (Fig. 1), cow peas (*Dolichossinensis* L.), pigeon peas (*Cajanuscajan* (L.) Huth), hyacinth bean (*Lablabpurpureus* (L.) Sweet), winged bean (*Psophocarpustetragonolobus* DC.) and the yam bean (*Pachyrhizuserosus* (L.) Urb.) (FAO 1994). Some of these beans, especially cow pea and pigeon pea, are sustainable and resilient crops, as they can be grown in poor-quality soils and are drought stress resistant (Varshney et al. 2009). This makes them particularly suitable for agricultural production where the growing of other crops would be difficult, if not impossible (Green et al. 2019).

**Figure 2.**
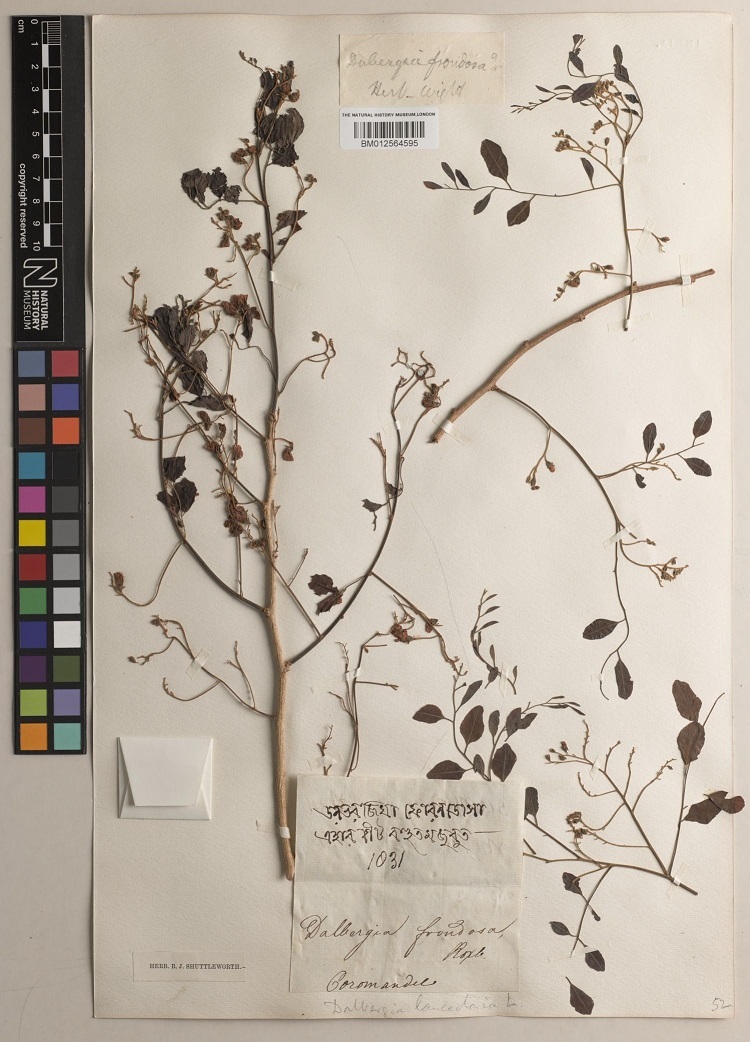
*Dalbergialanceolaria* L.f. (BM012564594).

The second case study focused on the conservation and sustainable use of rosewoods (*Dalbergia* L.f.) (Fig. 2) and padauk (*Pterocarpus* Jacq.). The timber from many species of *Dalbergia* and *Pterocarpus* is considered high-quality wood for construction, furniture, musical instruments and other decorative uses (Huang and Sun 2013). However, they are at risk of extinction due to illegal trade and habitat destruction (Cerutti et al. 2018, Green et al. 2019). Brazilian rosewood (*Dalbergianigra* (Vell.) Allemão ex Benth) is listed in CITES Appendix I and is assessed as Vulnerable on the IUCN Red List (Varty 1998, CITES 2021). All other *Dalbergia* species are listed in CITES Appendix II, as are three timber species of *Pterocarpus* species. CITES Appendix I species are threatened with extinction with trading permitted in exceptional circumstances, while CITES Appendix II species are potentially threatened with extinction unless trade is closely controlled (CITES 2022).

In addition to providing the specimen images and data, this paper describes the digitisation methodology, including the digitisation rates, photography, label data transcription and georeferencing protocols for the NHMUK specimens for both case study groups described above. We also summarise the geographic, taxonomic, temporal coverage and significant collectors of the NHMUK collections of *Dalbergia*, *Pterocarpus* and the Phaseolinae.

## General description

### Purpose

This article describes and analyses a digitised herbarium dataset published on the Natural History Museum's Data Portal (Lohonya et al. 2019) and on Zenodo with the project background.

## Project description

### Title

Defra ODA Legumes Project

### Personnel

Krisztina Lohonya, Robyn Crowther, Elizabeth Devenish, Louise Allan, Laurence Livermore, Jacek Wajer, Hillery Warner

### Design description

The collection was digitised with a mandate to create and publicly share a dataset of 10,000 herbarium specimens (images and metadata) to be used in scientific research into food security and timber production in ODA-listed countries. At the planning stage, we expected that around 75% of our digitised specimens would be from the ODA-listed countries (see the full list in the Appendix - ODA Countries), with 30% from the least developed to low and middle income countries, particularly in tropical Africa, but also in South East Asia and South America.

### Funding

This pilot project was made possible through the Department for Environment Food & Rural Affairs (DEFRA)-allocated, Official Development Assistance (ODA) funding. This aid money is distributed by the UK government in its “global efforts to defeat poverty, tackle instability and create prosperity in developing countries”. The Museum’s Digital Collections Programme supported the subsequent georeferencing work and writing of this paper.

## Sampling methods

### Study extent

**Collectors and collector coverage**

**Figure 3.**
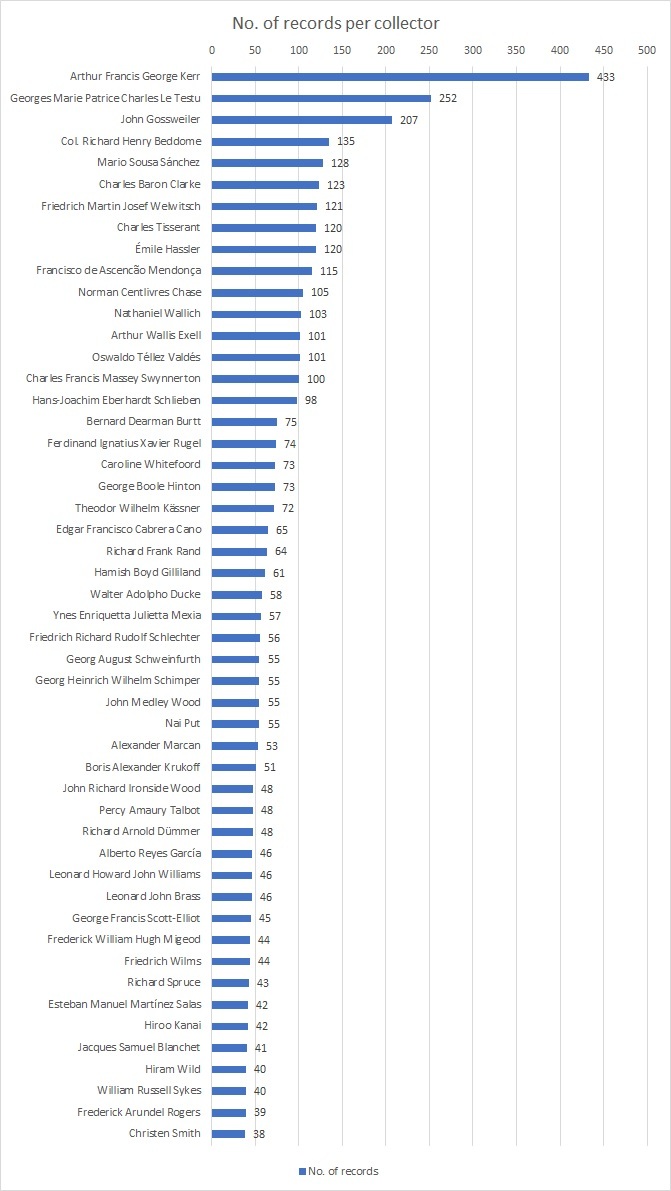
Counts of records from the top 50 collectors.

We were able to interpret and transcribe the collectors’ names from the majority of our specimen labels (10,879 out of 11,222). In 343 cases, the collector was either not identified on the label or we were unable to infer their identity either from their handwriting or using other information associated with their specimens. We recorded such collectors as ‘Anon.’ in our CMS (see Table 1) or 'Illegible' in cases where the collector's name was present, but we were unable to identify it. During the transcription stage, we identified 2,226 individuals who contributed to the collection. The most prolific collectors were Arthur Kerr (433 specimens), Georges Le Testu (252 specimens) and John Gossweiler (207 specimens) (see Fig. 3 and Table 2). Arthur Francis George Kerr predominantly collected in Thailand and surrounding countries. John Gossweiler’s collection mainly covers Angola and its neighbouring states. Georges Le Testu’s specimens were collected mostly in Gabon and the Central African Republic.

**Figure 4.**
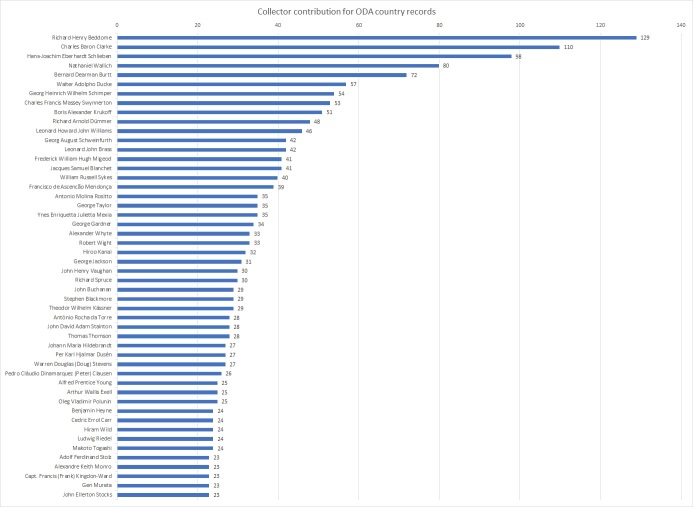
Collector's contribution for ODA-country records, where the 100% is the total number of records collected in ODA-listed countries.

Only 770 out of the 2,226 individuals identified during this project collected their specimens in the ODA-listed countries. The highest contributors were: Richard Beddome (130 specimens), Charles Clarke (110), Hans Schlieben (98) and Nathaniel Wallich (79). Below, you can see the percentage distribution of the 50 most active collector’s contributions. Beddome, Clarke and Wallich collected mostly in India and the surrounding countries. Schlieben’s collections are mainly from Tanzania and Madagascar (see Fig. 4 for more details).

### Step description

**Methodology - Databasing the collection**

**Figure 5.**
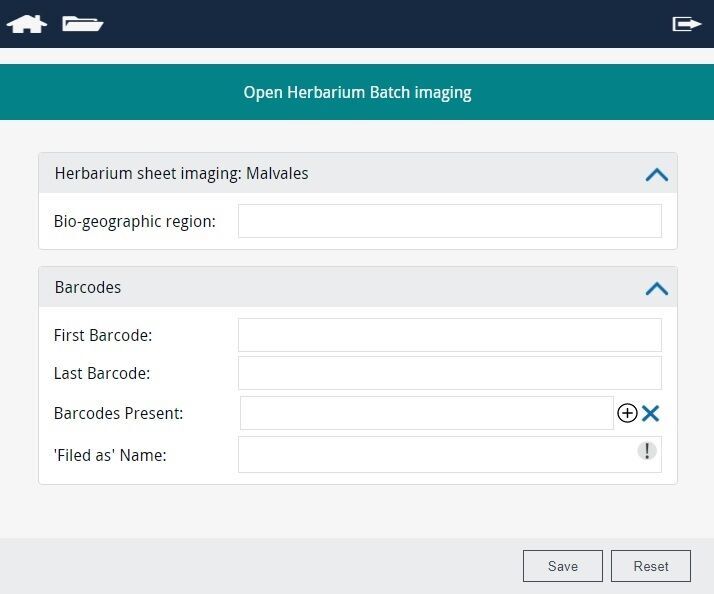
Web application for creating catalogue records, allowing us to create multiple records simultaneously and also record pre-existing barcodes without creating duplicate records in our CMS.

**Figure 6.**
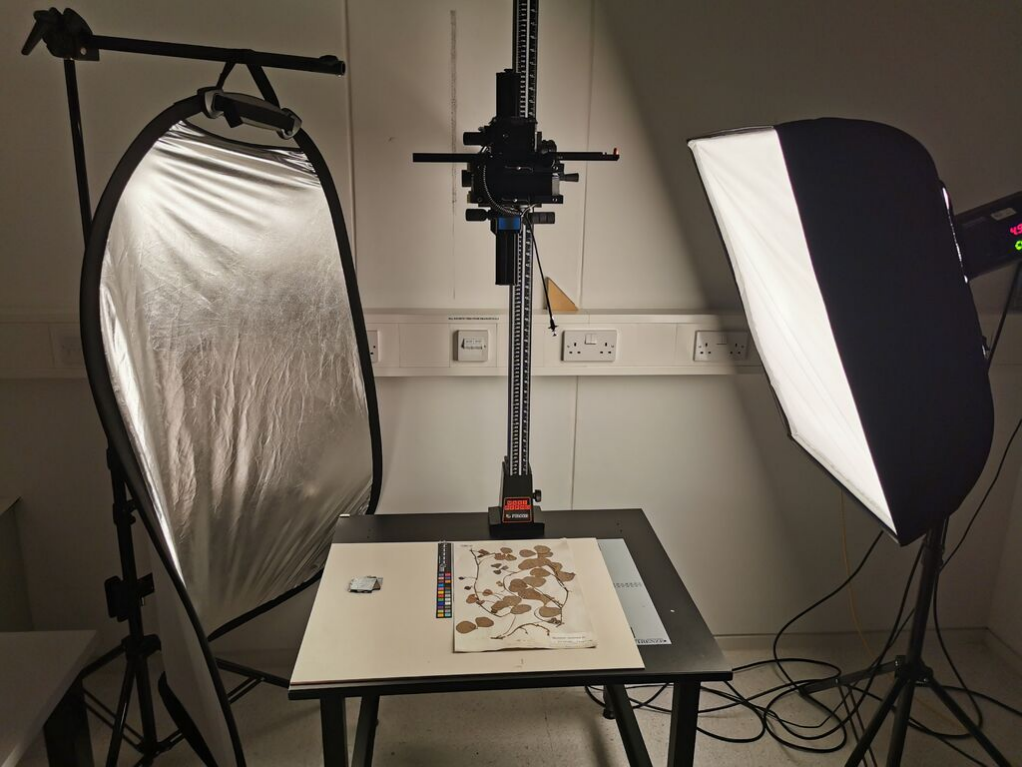
Imaging setup used for the project, consisting of a copystand with camera (centre), reflector (left) and flash (right).

The digitisation process was broken into several smaller steps to ensure a smooth workflow. First, the curator responsible for the Leguminosae family cleaned the existing database of the plant names in our collections management system (EMu) and ensured that all herbarium folders*^1^ had legible taxonomic and geographical information written on them to avoid any ambiguity during data capture. Having completed this digital pre-curation, we then proceeded to create the corresponding specimen records using a simple web-based application for mass digitisation (Fig. 5). In the first stage, we captured the bare minimum data associated with each specimen to create the inventory or ‘stub’ records, consisting of a unique linear barcode identifier for each specimen, the taxonomic name under which the specimen is filed in the collection and the geographic region under which it is curated (see Geographical regions in the Appendix). While our herbarium has a mix of expert identifications and assumed identifications based on filing names/placement in collection, we have not differentiated this in the current dataset for this project. The specimens were then imaged using a Leaf Aptus-II 10(LI300321)/Large Format Digital Back 80 MP camera setup (Fig. 6). Capture One version 10 for Mac was also used for image editing.

In the next phase, we transcribed the label information associated with each specimen from the images generated during the stub-recording stage. We adopted an existing transcription protocol used by the curation team at the BM Herbarium to standardise the resulting data, with set marking for missing information (see Table 1). To make the transcription process even more efficient, we divided the entire dataset into smaller sections consisting of the records corresponding to one genus and its geographic regions - this is based on the physical filing data from the folders containing the sheets. In the first step, we only transcribed the collector’s name, the collection date and the collection number associated with each specimen. In the next phase, we sorted all data by the collector and the collection date and then transcribed the collection locality from each label. This approach ensured that records for specimens from different taxonomic groups collected by the same people from the same or nearby localities were clustered together, which made the transcription process more streamlined.

During georeferencing, we followed the NHMUK’s georeferencing protocols and geographical standards (Penn et al. 2017).

During the funded part of the project (November 2018 – March 2019), we stub-recorded and imaged 11,222 specimens of *Dalbergia*, *Pterocarpus* and 51 genera in the subtribe Phaseolinae and their relatives, but in that period, we only managed to transcribe the label information and georeference the records of *Dalbergia* and *Pterocarpus*. After the initial project completion, during 2020–2021, we completed the transcription and country-level georeferencing for all remaining records. Just like in the pilot project, we georeferenced in two stages: first, we worked out the current name of the country of collection for each specimen, as many of them came from the territories that have changed their names or their boundaries have shifted, then we completed georeferencing with coordinates for each location. In the initial phase of the pilot, when we fully transcribed and georeferenced all records of *Dalbergia* and *Pterocarpus*, we achieved a rate of approximately 86 transcribed and georeferenced records per day per person (Table 3). When we did full transcription in the 2020/2021 period, with georeferencing efforts scaled down to the country-level only, we increased our transcription rate to ca. 115 records per day. It may seem that georeferencing in the second scenario was incomplete, but in this way, we have relatively quickly produced a fully transcribed and searchable dataset with all records having their collecting localities correctly interpreted in the modern geographical sense, making any future georeferencing work more straightforward.

We were able to assign the country-level data to 10,857 out of the total number of 11,222 records. The collecting locality for the remaining records was either lost (unknown for at least 32 specimens) or exact georeferencing was not possible due to the ambiguity of the provenance data. This problem is particularly prevalent in older herbarium sheets which usually have rather vague locality information associated with them. For example, the specimens collected by Christen Smith during Captain Tuckey’s expedition to Congo in 1816 have no precise locality associated with them other than ‘Congo’. Without any additional data from Smith's diary or from Tuckey’s charts of the River Congo, it is not possible to determine if these specimens were collected in the modern-day Angola, the Democratic Republic of the Congo or the Republic of the Congo, all three countries bordering the course of the expedition undertaken by these two explorers. In other instances, exact georeferencing was not possible because the territory listed on the label does not exist anymore or it currently covers an area of many different countries. For example, the locality “New Grenada” without any additional information is not precise enough to be assigned to just one specific country, as this historical Viceroyalty of the Spanish Empire in the northern part of South America covered what is now parts of Brazil, Colombia, Guyana, Ecuador, Panama, Peru, Suriname, Trinidad and Tobago and Venezuela.

**Digitisation rates**

The collection was digitised by three digitisers: one full-time and two part-time. The initial steps included curatorial checks, barcoding and inventory record creation. The imaging part of the digitisation consisted of image capture, file transfer, upload and record linkage in our collection management system (CMS). During the stub-recording phase, we generated, on average, 400 records per day per person. During the imaging part of the project, we photographed ca. 380 specimens per day per person. Our imaging rates were lower than stub-recording because many of our specimens required taking more than one image due to having their provenance notes written at the back of the sheets or having their collection labels and identification labels covering each other (Table 3).

With our initial approach for comprehensive data transcription, used only for the *Dalbergia* and *Pterocarpus* dataset (November 2018 to March 2019), we were able to interpret and fully georeference, on average, 86 records per day per person. In the second phase of the project (November 2020 to April 2021), during which we transcribed the collection data for all the remaining specimens of the Phaseolineae subtribe and their relatives, but with georeferencing of the collecting sites scaled down to a country level only, we were able to complete, on average, 115 records per day (Table 4).

## Geographic coverage

### Description

Most of the specimens digitised as part of this pilot have been georeferenced at least to a country level. We were unable to assign modern country names to 365 out of the total 11,222 records, all of which had either incomplete or ambiguous geographical information associated with them.

**Figure 7.**
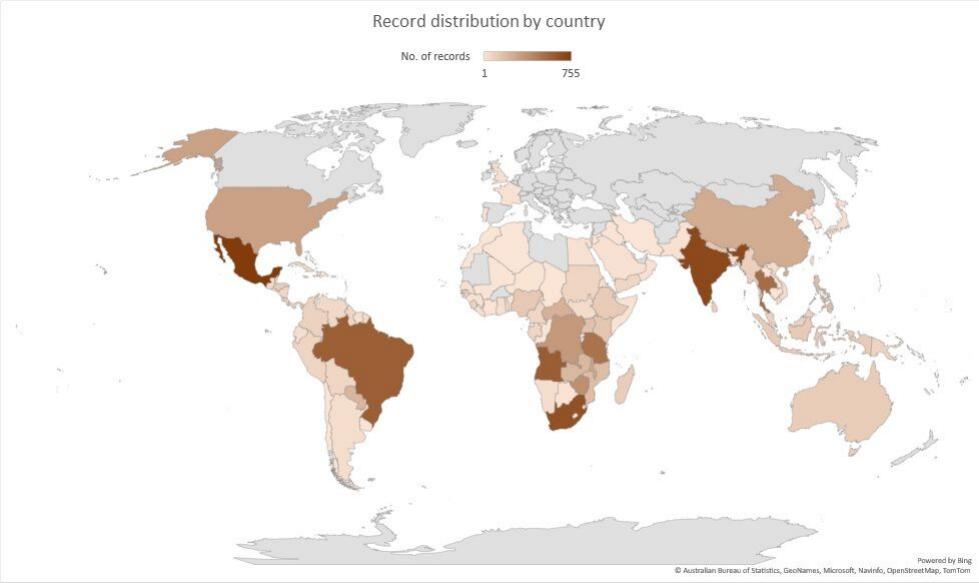
Record distribution by country.

We established that the remaining 10,857 specimens were collected from at least 142 countries around the world (Fig. 7, Suppl. material 3).

The countries with the most records are Mexico (755), India (699), South Africa (658), Angola (607) and Brazil (599) (see the full list: Suppl. material 1).

**Figure 8.**
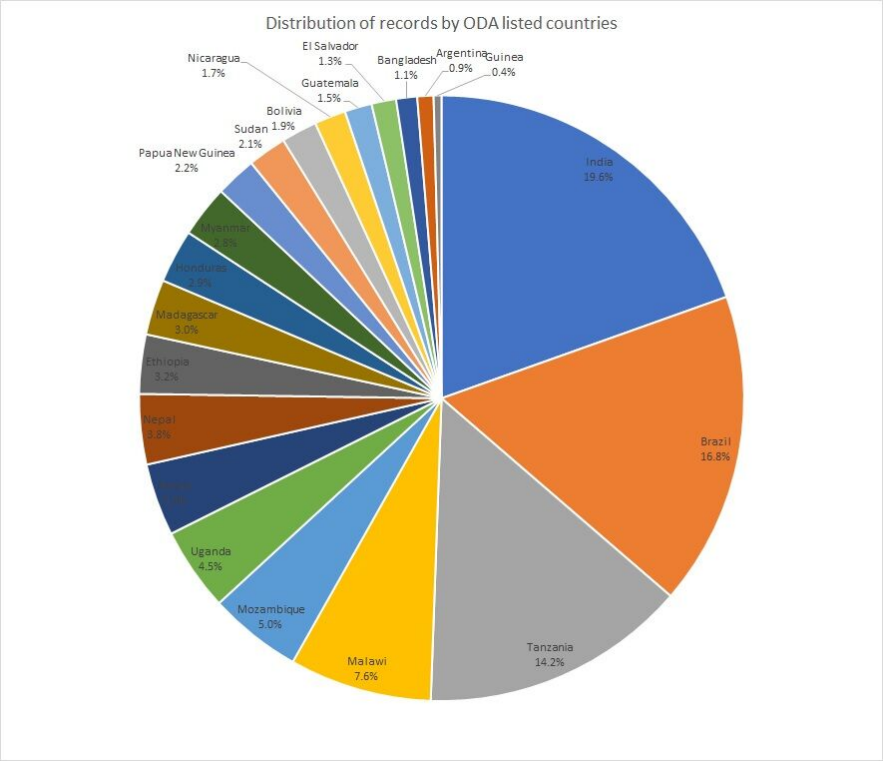
Distribution of records by ODA-listed countries, where 100% is the total number of records collected in ODA-listed countries.

The coverage of the ODA listed countries was of a particular interest to us. When only placing the ODA countries in the chart (Fig. 8), it is clear that slightly more than 50% of our records are from the following three countries: India (19.6%), Brazil (16.8%) and Tanzania (14.2%). There is a particularly low representation from Guinea (0.4%).

## Taxonomic coverage

### Description

**Taxonomic Scope**

- *Dalbergia* L.f. (global tropical trees, shrubs & lianas, includes rosewoods [*Fabaceae:Faboideae*])
- *Pterocarpus* Jacq. (pantropical tree genus aka Padauk, [*Fabaceae:Faboideae*])
- Phaseolinae [Fabaceae: Faboideae] genera:
  - *Alistilus*
  - *Condylostylis*
  - *Decorsea*
  - *Dipogon*
  - *Dolichopsis*
  - *Dolichos*
  - *Dysolobium*
  - *Kerstingiella*
  - *Lablab*
  - *Macroptilium*
  - *Macrotyloma*
  - *Minkelersia*
  - *Neorautanenia*
  - *Nesphostylis*
  - *Otoptera*
  - *Oxyrhynchus*
  - *Phaseolus*
  - *Physostigma*
  - *Psophocarpus*
  - *Ramirezella*
  - *Spathionema*
  - *Sphenostylis*
  - *Strophostyles*
  - *Vatovaea*
  - *Vigna*
- Other Fabaceae genera (Tribe or Subtribe):
  - *Cyclolobium* (Brongniartieae)
  - *Adenodolichos* (Cajaninae)
  - *Atylosia* (Cajaninae)
  - *Bolusafra* (Cajaninae)
  - *Cajanus* (Cajaninae)
  - *Carrissoa* (Cajaninae)
  - *Dunbaria* (Cajaninae)
  - *Eriosema* (Cajaninae)
  - *Flemingia* (Cajaninae)
  - *Paracalyx* (Cajaninae)
  - *Rhynchosia* (Cajaninae)
  - *Centrolobium* (Dalbergieae)
  - *Machaerium* (Dalbergieae)
  - *Paramachaerium* (Dalbergieae)
  - *Platypodium* (Dalbergieae)
  - *Steinbachiella* (Dalbergieae)
  - *Tipuana* (Dalbergieae)
  - *Vatairea* (Dalbergieae)
  - *Vataireopsis* (Dalbergieae)
  - *Eminia* (Glycininae)
  - *Glycine* (Glycininae)
  - *Pachyrhizus* (Glycininae)
  - *Pseudeminia* (Glycininae)
  - *Pseudovigna* (Glycininae)
  - *Phylloxylon* (Indigofereae)
  - *Dalbergiella* (Millettieae)

**Figure 9.**
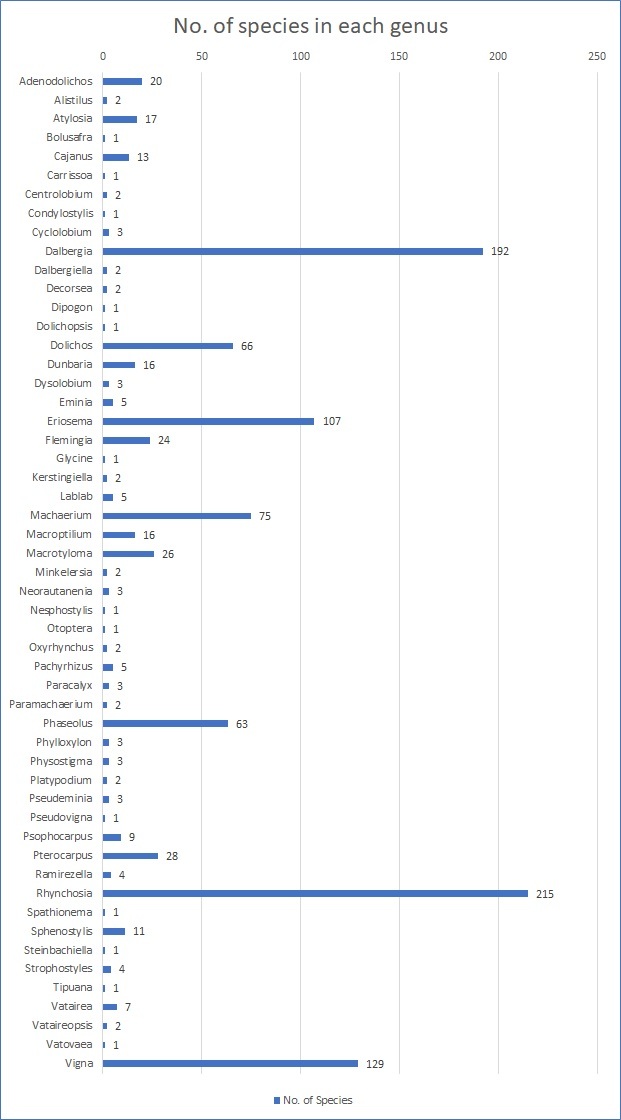
Count of species from each genus.

Our set includes the data transcribed from the labels of 11,208 herbarium specimens of the legume genera *Dalbergia* L.f. and *Pterocarpus* Jacq. and from the 51 genera of the subtribe Phaseolinae and their relatives in the tribes Brongniartieae, Dalbergieae, Indigofereae, Millettieae and Phaseoleae. In total, we have digitised 192 species of *Dalbergia* and 28 species of *Pterocarpus* currently found in our collection. In the remaining group, the three genera of which we have the most species in our collection are: *Rhynchosia* (215), *Vigna* (129) and *Eriosema* (107) (Fig. 9).

## Temporal coverage

### Notes

**Figure 10.**
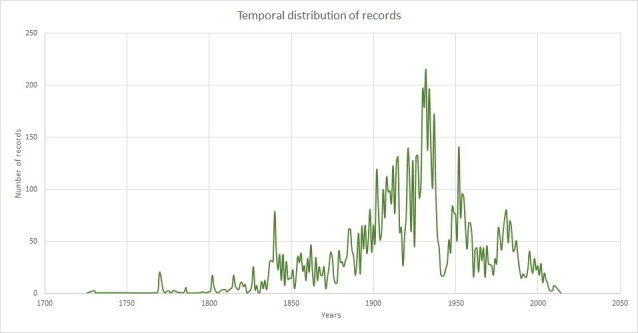
No. of records per year.

From the 11,222 records in our dataset, we could identify temporal qualifiers for 9271 records. From these records, the peak decade of collection was the 1930s (1,493 records or 16.1%), with almost half (4,583 specimens or 49.43%) collected between 1900 and 1950 (Fig. 10). This peak can be attributed to three of our most prolific collectors: Arthur Kerr, John Gossweiler and Georges Le Testu, all of whom were most active in the 1930s. There is a significant dip around the 1940s which is likely due to the Second World War. The oldest specimen (BM013713473) was collected by Mark Catesby (1683-1749) in the Bahamas in 1726. A total of 1,951 specimens lacked any information about the collection date. These were recorded in our CMS as 'sin. dat.' (Table 1).

There are also two smaller collecting peaks around the 1840s and 1980s. The first peak can be attributed to Ferdinand Rugel, who collected in the Americas and Carl Zeyher, who collected in South Africa. The second peak can be attributed to Caroline Whitefoord, Edgar Cabrera Cano, Mario Sousa Sánchez and colleagues collecting extensively in Central America.

## Collection data

### Collection name

The Natural History Museum General Herbarium

### Collection identifier

BM

## Usage licence

### Usage licence

Creative Commons Public Domain Waiver (CC-Zero)

## Data resources

### Data package title

Defra ODA Legumes Project

### Resource link

https://doi.org/10.5281/zenodo.7274404

### Alternative identifiers

https://doi.org/10.5519/0073006

### Number of data sets

1

### Data set 1.

#### Data set name

Defra ODA Legumes Project

#### Description

The dataset consists of 11,222 records of digitised herbarium specimens. For each specimen, the species name, locality, collection date, collector and collection number are recorded.

**Data set 1.** 

Column label	Column description
dwc:basisOfRecord	The specific nature of the data record.
dwc:occurrenceID	An identifier for the Occurrence (as opposed to a particular digital record of the occurrence). In the absence of a persistent global unique identifier, construct one from a combination of identifiers in the record that will most closely make the occurrenceID globally unique.
_id	Equivalent of a primary key.
dwc:recordNumber	An identifier given to the Occurrence at the time it was recorded. Often serves as a link between field notes and an Occurrence record, such as a specimen collector's number.
dwc:institutionCode	The name (or acronym) in use by the institution having custody of the object(s) or information referred to in the record.
dwc:collectionCode	The name, acronym, coden or initialism identifying the collection or dataset from which the record was derived.
dwc:catalogNumber	An identifier (preferably unique) for the record within the dataset or collection.
dwc:otherCatalogNumbers	A list (concatenated and separated) of previous or alternative fully qualified catalogue numbers or other human-used identifiers for the same Occurrence, whether in the current or any other dataset or collection.
dwc:recordedBy	A list (concatenated and separated) of names of people, groups or organisations responsible for recording the original Occurrence. The primary collector or observer, especially one who applies a personal identifier (recordNumber), should be listed first.
dwc:family	The full scientific name of the family in which the taxon is classified.
dwc:higherClassification	A list (concatenated and separated) of taxa names terminating at the rank immediately superior to the taxon referenced in the taxon record.
dwc:order	The full scientific name of the order in which the taxon is classified.
dwc:genus	The full scientific name of the genus in which the taxon is classified.
dwc:verbatimLocality	The original textual description of the place.
dwc:specificEpithet	The name of the first or species epithet of the scientificName.
dwc:scientificNameAuthorship	The authorship information for the scientificName formatted according to the conventions of the applicable nomenclaturalCode.
dwc:higherGeography	A list (concatenated and separated) of geographic names less specific than the information captured in the locality term.
dwc:continent	The name of the continent in which the Location occurs.
dwc:country	The name of the country or major administrative unit in which the Location occurs.
dwc:eventDate	The date-time or interval during which an Event occurred. For occurrences, this is the date-time when the event was recorded. Not suitable for a time in a geological context.
dwc:locality	The specific description of the place.
dwc:stateProvince	The name of the next smaller administrative region than country (state, province, canton, department, region etc.) in which the Location occurs.
dwc:decimalLatitude	The geographic latitude (in decimal degrees, using the spatial reference system given in geodeticDatum) of the geographic centre of a Location. Positive values are north of the Equator, negative values are south of it. Legal values lie between -90 and 90, inclusive.
dwc:decimalLongitude	he geographic longitude (in decimal degrees, using the spatial reference system given in geodeticDatum) of the geographic centre of a Location. Positive values are east of the Greenwich Meridian, negative values are west of it. Legal values lie between -180 and 180, inclusive.
dwc:georeferenceProtocol	A description or reference to the methods used to determine the spatial footprint, coordinates and uncertainties.
dwc:county	The full, unabbreviated name of the next smaller administrative region than stateProvince (county, shire, department, etc.) in which the Location occurs.
dwc:minimumElevationInMetres	The lower limit of the range of elevation (altitude, usually above sea level), in metres.
dwc:georeferenceSources	A list (concatenated and separated) of maps, gazetteers or other resources used to georeference the Location, described specifically enough to allow anyone in the future to use the same resources.
dwc:infraspecificEpithet	The name of the lowest or terminal infraspecific epithet of the scientificName, excluding any rank designation.
dwc:island	Recommended best practice is to use a controlled vocabulary such as the Getty Thesaurus of Geographic Names.
dwc:islandGroup	The name of the island group in which the Location occurs.
dwc:scientificName	The full scientific name, with authorship and date information, if known. When forming part of an Identification, this should be the name in the lowest level taxonomic rank that can be determined. This term should not contain identification qualifications, which should instead be supplied in the IdentificationQualifier term.
dwc:taxonRank	The taxonomic rank of the most specific name in the scientificName.
dwc:maximumElevationInMetres	The upper limit of the range of elevation (altitude, usually above sea level), in metres.
dwc:habitat	A category or description of the habitat in which the Event occurred.
dwc:typeStatus	A list (concatenated and separated) of nomenclatural types (type status, typified scientific name, publication) applied to the subject.
dwc:waterBody	The name of the water body in which the Location occurs.
dwc:geodeticDatum	The ellipsoid, geodetic datum or spatial reference system (SRS) upon which the geographic coordinates given in decimalLatitude and decimalLongitude are based.

## Additional information

### Discussion

The project had a tight timeframe: we had a four-month period to create inventory records, image and transcribe an estimated 10,000 specimens. During the project, we recorded 138 person days of digitiser effort, split amongst three digitisers. The creation of the stub-records and imaging was successfully completed within this period, but the transcription of such a large number of specimens required more time. The digitisation rate for creating inventory records and the specimen imaging met our expectations of creating an average of 400 records and imaging ca. 380 specimens per day (Table 2). The transcription rates and quality were good: two out of the three digitisers involved in the project had no previous experience in transcribing collecting information associated with herbarium specimens, although both were already familiar with the labels on the specimens in our zoological collection which are usually less detailed. Their transcription rates, however, were comparable to those of the curator in charge of the legume collection who has an extensive knowledge of the corresponding collectors, their handwriting styles and their collecting localities.

When planning the project, we expected that approximately 75% of our collection will be from the ODA-listed countries. The original estimate was based on the collections of RBGK and we assumed that the collection localities would be similar. Following digitisation and data capture, we found that the ODA-listed countries covered only 31.8% of the NHM collection. We discovered that we have a large number of specimens from Mexico, South Africa, Angola and Thailand, all of which are not on the ODA list. The discrepancy between the original estimate of ~ 75% of material from ODA-listed countries versus the actual figure of 31.8% is likely due to historical differences in our collections and acquisitions compared with RBGK. While we expected some level of difference, this was greater than we anticipated. As collections are organised by taxon, it is hard to make predictions on collection locality without extensive random sampling or understanding systematic patterns like those seen with collectors. This is a factor we hope to better estimate in future digitisation projects.

Our transcription efforts were focused on only the five most crucial aspects of the collecting information associated with herbarium specimens. These were: the taxonomy (i.e. the name under which the specimen is filed in the herbarium, not just the name on the label which is sometimes different), the name of the person or the people who collected the specimen, the collection number under which it was recorded, the collection date or date ranges and, finally, the country of collection and the precise details of the collecting site. These data are vital, not only for the corresponding records to be findable in our Collections Management System, but to better inform us about any temporal or spatial changes to global biodiversity due to climate change or through any other factors. The labels associated with the herbarium specimens, however, often contain a much wider range of information than these five key elements, most commonly habitat description, vernacular names, local uses and occasionally even a cooking recipe if the plant is eaten by native people. We did not transcribe this type of information as it was not required for our project and we have to prioritise data capture, based on project requirements due to the scale of our collections. However, it can still be extracted from the associated images and might be of use to all sorts of other research projects, such as ecological or ethnobotanical studies.

As is typical for older natural history specimens, some of the transcribed data include historical regions or countries. While we have general point-radius coordinates with high uncertainty for modern countries, we do not have this for historical regions. We recognise that this is potentially valuable data and could lead to inaccurate interpretations (e.g. wrongly intepreting the locality "Congo" as the modern day Democratic Republic of the Congo). This issue has been previously identified as a community challenge by Marcer et al. (2020): "*there is a lack of publicly shared, global, hierarchical, time-aware, community-vetted geographical directories, gazetteers*".

An interesting, but perhaps unsurprising, finding is that our collection seems to be strongly male dominated. There are only two women (Caroline Whitefoord and Ynes Mexia) in the list of our top 50 plant collectors (Table 4) and they are not close to the most prolific collectors. We identified more women in the rest of our records, but their contribution is, on average, less than 25 specimens per person in the dataset consisting of more than 10,000 specimens. In contrast, the top five male collectors contributed 10% of our collection. It would be useful to check in the future if this trend is replicated throughout the rest of our entire herbarium collection, which consists of an estimated 5 million specimens or if this is unique to the collections in this project.

Overall, we consider the project successful given we had four months and three members of staff to digitise 11,222 herbarium specimens. The Covid-19 induced lockdown, despite its negative impact on many aspects of our private and professional lives, was also ironically rather fortuitous as it enabled us to transcribe and georeference all the digital records generated though this project from our homes. This proves that digitising the herbarium specimens at an inventory level first and then enhancing these records with more detailed or project-specific data some time later might be the way forward for any future large-scale digitisation endeavours which will require vast numbers of specimens to be processed as quickly and as effectively as possible. We also hope that the results of our work presented here will help other herbarium professionals better plan for their own digitisation projects.

Finally, as part of the publication process, this manuscript failed the initial technical validation using the original dataset which is a live version hosted on our insitutional data portal (Scott et al. 2019), synchronised with all the specimen records in our CMS. In order to meet the technical validation requirements and adhere to Darwin Core (Wieczorek et al. 2012, Darwin Core maintenance group. Biodiversity Information Standards (TDWG) 2018), we had to make some changes that were not supported by either our data portal or our CMS or were due to incompatibilities in data transfer between the two systems (e.g. conversion of dates to ISO 8601). We highlight this as some of these issues are not easy fixes and are likely to prevent the sharing of data using these systems without additional editing. We fixed some legacy data quality issues (e.g. variations of country name, over accuracy of latitude and longitude), but were unable to check and correct historical, non-standardised georeferencing protocol data. This particular issue is recognised as one of many broad quality issues in georeferencing (Marcer et al. 2020). Some issues, like created and modified data being stored in milliseconds from the UNIX epoch rather than ISO 8601 timestamps, but are planned to be fixed in future releases of the NHM Data Portal.

### Definitions

- **CMS**: Collections management system, in reference to the NHMUK’s CMS which is currently Axiell's EMu.
- **Data Portal**: The NHMUK’s open access, open source data portal which provides an online human and machine-readable interface to the NHMUK’s collections. The collection data come from our CMS.
- **GBIF**: Global Biodiversity Information Facility. Global aggregator for biodiversity data. NHMUK provides data to GBIF via the Data Portal.
- **Inventory record**: the minimum information digital record of a herbarium specimen consisting of a unique barcode identifier, the taxonomic name under which the specimen is filed in the collection and the herbarium region under which it is curated.
- **NHMUK**: Natural History Museum, London. Partner on this project.
- **ODA**: Official Development Assistance. UK government aid programme targeting the economic development and welfare of developing countries.
- **RBGK**: Royal Botanic Gardens, Kew. Lead on this project.
- **RBGE**: Royal Botanic Garden Edinburgh. Partner on this project.

### Appendix

**ODA-listed Countries**

**African**: Guinea, Ethiopia, Sudan, Kenya, Uganda, Tanzania, Mozambique, Malawi and Madagascar.

**Asian**: Bangladesh, Myanmar, Nepal, New Guinea and India.

**Southern and Central American**: Guatemala, Honduras, El Salvador, Nicaragua, Bolivia, Argentina and Brazil.

The following least-developed or low-income countries are official partners in GBIF: Benin, Central African Republic, DRC Congo, Guinea, Kenya, Madagascar, Malawi, Mauritania, Niger, South Sudan, Tanzania, Togo, Uganda and Zimbabwe.

**Geographical regions**

All plant genera at the BM Herbarium are curated using a unique system of 26 biogeographical regions:

- Europe
- Western Asia
- North Asia
- Japan
- China
- India
- Indochina
- Malesia
- West African Islands
- North Africa
- Tropical Africa
- South Africa
- South African Islands
- Madagascar
- Mascarenes
- Australia
- New Zealand
- New Caledonia
- Polynesia
- North America
- Central American Continent
- West Indies
- Tropical South America
- Brazil
- Temperate South America
- Antarctica

## Supplementary Material

0AF43275-BCA1-5ADE-9160-096912B0BE56 10.3897/BDJ.10.e94939.suppl1 Supplementary material 1 Full country list Data type Geographical Brief description A list of all countries represented in our dataset with the corresponding numbers of specimens. File: oo_744237.csv https://binary.pensoft.net/file/744237 Krisztina Lohonya

A95111F7-DF12-50A3-B561-AB6BA25E0F70 10.3897/BDJ.10.e94939.suppl2 Supplementary material 2 Collector's list Data type collector, humanities Brief description A list of all collectors represented in our dataset and the numbers of the specimens contributed by them. File: oo_744239.csv https://binary.pensoft.net/file/744239 Krisztina Lohonya

BC4D70A9-2705-548C-A296-86F321FB47D5 10.3897/BDJ.10.e94939.suppl3 Supplementary material 3 Number of records per country Data type bar chart Brief description A bar chart representation of the number of records from each country. File: oo_764824.jpg https://binary.pensoft.net/file/764824 Krisztina Lohonya

## Figures and Tables

**Table 1.** Abbreviations used when information is missing or unknown.

**Collector**	**Collection number**	**Collection date**	**Locality**
Anon.	s.n.	sin. dat.	sin. loc.

**Table 2.** List of top 50 collectors by the number of records in the ODA-digitised section of the herbarium (full list available in the Suppl. material 2).

	**Wikidata Q Code**	**Collector Name**	**No. of records**
1	Q4798733	Arthur Francis George Kerr	433
2	Q55684213	Georges Marie Patrice Charles Le Testu	252
3	Q15727183	John Gossweiler	207
4	Q2985757	Richard Henry Beddome	135
5	Q56167901	Mario Sousa Sánchez	128
6	Q2659116	Charles Baron Clarke	123
7	Q78609	Friedrich Martin Josef Welwitsch	121
8	Q21340735	Charles Tisserant	120
9	Q116720	Émile Hassler	120
10	Q4290420	Francisco de Ascencão Mendonça	115
11	Q108197157	Norman Centlivres Chase	105
12	Q730310	Nathaniel Wallich	103
13	Q2865378	Arthur Wallis Exell	101
14	Q5651733	Oswaldo Téllez Valdés	101
15	Q5077783	Charles Francis Massey Swynnerton	100
16	Q21337647	Hans-Joachim Eberhardt Schlieben	98
17	Q2897760	Bernard Dearman Burtt	75
18	Q21607532	Ferdinand Igatius Xavier Rugel	74
19	Q21395774	Caroline Whitefoord	73
20	Q55532773	George Boole Hinton	73
21	Q108197234	Theodor Wilhelm Kässner	72
22	Q108197361	Edgar Francisco Cabrera Cano	65
23	Q55455995	Richard Frank Rand	64
24	Q21340660	Hamish Boyd Gilliland	61
25	Q2601698	Walter Adolpho Ducke	58
26	Q2600470	Ynes Enriquetta Julietta Mexia	57
27	Q62278	Friedrich Richard Rudolf Schlechter	56
28	Q63126	Georg August Schweinfurth	55
29	Q72899	Georg Heinrich Wilhelm Schimper	55
30	Q1701052	John Medley Wood	55
31	Q108197317	Nai Put	55
32	Q108197276	Alexander Marcan	53
33	Q5732192	Boris Alexander Krukoff	51
34	Q5933549	John Richard Ironside Wood	48
35	Q2043550	Percy Amaury Talbot	48
36	Q3430474	Richard Arnold Dümmer	48
37	Q21607143	Alberto Reyes García	46
38	Q21612729	Leonard Howard John Williams	46
39	Q6525437	Leonard John Brass	46
40	Q5877823	George Francis Scott-Elliot	45
41	Q108197333	Frederick William Hugh Migeod	44
42	Q5503949	Friedrich Wilms	44
43	Q1349394	Richard Spruce	43
44	Q13501872	Esteban Manuel Martínez Salas	42
45	Q21517258	Hiroo Kanai	42
46	Q3159964	Jacques Samuel Blanchet	41
47	Q5792958	Hiram Wild	40
48	Q6167727	William Russell Sykes	40
49	Q21607328	Frederick Arundel Rogers	39
50	Q1078398	Christen Smith	38

**Table 3.** Digitisation rates, 2018–2019.

**Date**		**Average number of inventory records per day**	**Average number of specimens imaged per day**	**Average number of records transcribed and georeferenced per day**
November	2018	297	n/a	n/a
December	2018	434.5	610.5	n/a
January	2019	412.5	429	82.5
February	2019	385	280.5	104.5
March	2019	374	209	71.5

**Table 4.** Digitisation (Transcription) rates, 2020–2021. *We had minor problems with our CMS system at that time.

**Date**	**Number of specimens**	**Number of working days**	**Average number of specimens transcribed per day**
November 2020*	1941	21.6	89.9
December 2020	1317	11.65	113
January 2021	1714	13.75	124.7
February 2021	1396	12.3	113.5
March 2021	2002	16.85	118.8
April 2021	638	5	127.6

## Footnotes

*1 The majority of the NHMUK herbarium sheets are stored in cabinets with shelves. The sheets are organised in folders and arranged by genera, geographic regions and species. The order of species within the herbarium is mixed with some sections using historical treatments/monographs, while recently-curated sections have switched to using alphabetical organisation. There was a mix of sheet organisation in the sections included in this project.
