# Supplementary figures and images for: Digitisation of the Natural History Museum’s collection of Dalbergia, Pterocarpus and the subtribe Phaseolinae (Fabaceae, Faboideae)
Source: Biodivers Data J. 2022 Nov 14;10:e94939. doi: 10.3897/BDJ.10.e94939 (PMC9836434; doi:10.3897/BDJ.10.e94939)

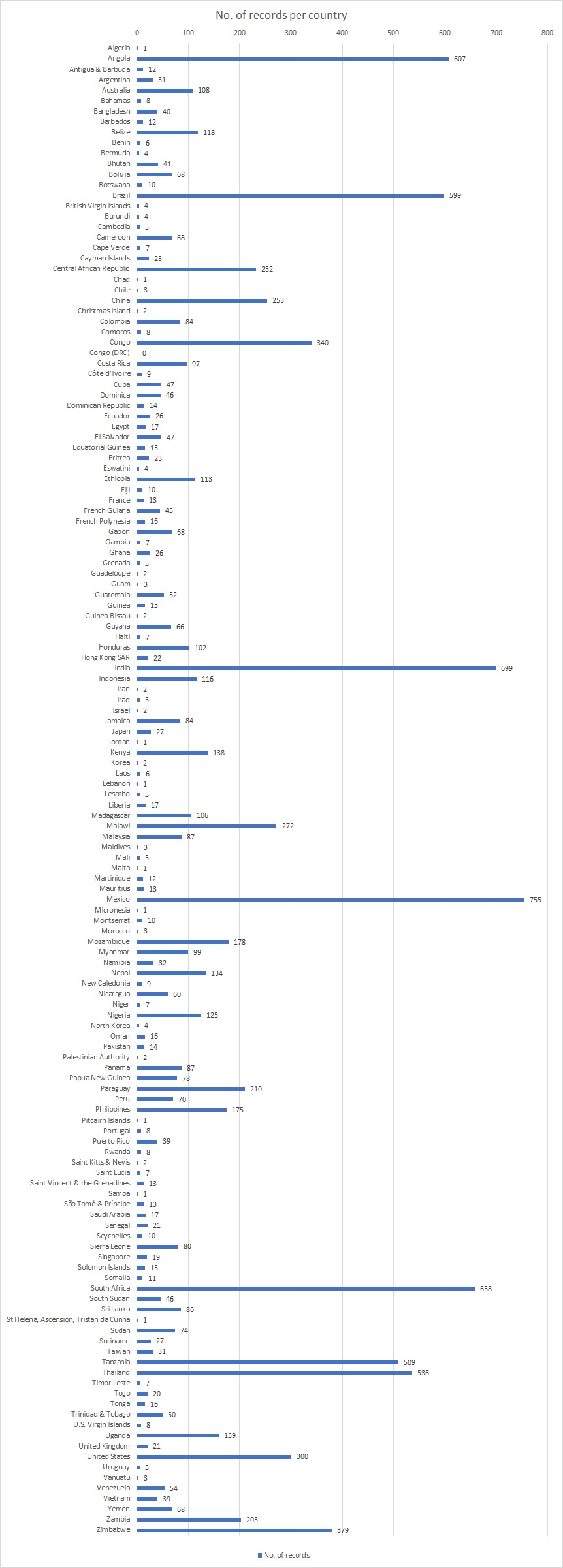

Supplement: Supplementary material 3 — Number of records per country [file bdj-10-e94939-s003.jpg]
